# Chemical suppression of harmaline-induced body tremor yields recovery of pairwise neuronal coherence in cerebellar nuclei neurons

**DOI:** 10.3389/fnsys.2023.1135799

**Published:** 2023-05-11

**Authors:** Yuval Baumel, Hagar Grazya Yamin, Dana Cohen

**Affiliations:** The Leslie and Susan Gonda Multidisciplinary Brain Research Center, Bar-Ilan University, Ramat-Gan, Israel

**Keywords:** cerebellum, tremor, oscillations, harmaline, synchrony, chronic recordings

## Abstract

Neuronal oscillations occur in health and disease; however, their characteristics can differ across conditions. During voluntary movement in freely moving rats, cerebellar nuclei (CN) neurons display intermittent but coherent oscillations in the theta frequency band (4–12 Hz). However, in the rat harmaline model of essential tremor, a disorder attributed to cerebellar malfunction, CN neurons display aberrant oscillations concomitantly with the emergence of body tremor. To identify the oscillation features that may underlie the emergence of body tremor, we analyzed neuronal activity recorded chronically from the rat CN under three conditions: in freely behaving animals, in harmaline-treated animals, and during chemical suppression of the harmaline-induced body tremor. Suppression of body tremor did not restore single neuron firing characteristics such as firing rate, the global and local coefficients of variation, the likelihood of a neuron to fire in bursts or their tendency to oscillate at a variety of dominant frequencies. Similarly, the fraction of simultaneously recorded neuronal pairs oscillating at a similar dominant frequency (<1 Hz deviation) and the mean frequency deviation within pairs remained similar to the harmaline condition. Moreover, the likelihood that pairs of CN neurons would co-oscillate was not only significantly lower than that measured in freely moving animals, but was significantly worse than chance. By contrast, the chemical suppression of body tremor fully restored pairwise neuronal coherence; that is, unlike in the harmaline condition, pairs of neurons that oscillated at the same time and frequency displayed high coherence, as in the controls. We suggest that oscillation coherence in CN neurons is essential for the execution of smooth movement and its loss likely underlies the emergence of body tremor.

## 1. Introduction

Neuronal oscillations are broadly distributed throughout the brain; nonetheless, the role they play in health and disease and how their characteristics evolve with disease progression remain elusive (Sharott et al., 2005; Leventhal et al., 2012; Basar, 2013; Basar and Guntekin, 2013; Matzner et al., 2016; Giovanni et al., 2017; Kuo et al., 2019; Mizrahi-Kliger et al., 2020). The movement production network provides an excellent model for studying oscillations in health and disease because brain oscillations play a prominent role in the performance of smooth and accurate movements in healthy individuals (for physiological tremor see Schnitzler et al., 2006; Williams et al., 2010), and network malfunction disrupts overt movements (Hutchison et al., 2004; Schnitzler et al., 2006; Mallet et al., 2008; Crowell et al., 2012; Kondylis et al., 2016; Bhatia et al., 2018; Schnitzler et al., 2018; Dubbioso and Tomasevic, 2019).

Essential tremor (ET) is one of the most prevalent forms of malfunction in the movement production network, which is expressed as uncontrolled, rhythmic movements primarily of the upper limbs and the head (Critchley, 1949; Critchley, 1972; Deuschl et al., 1998). Over the years, a plethora of evidence has implicated the olivocerebellar system with ET (Lamarre et al., 1971; Lamarre and Weiss, 1973; Llinas and Volkind, 1973; Weiss and Pellet, 1981; Welsh, 1998; Koster et al., 2002; Beitz and Saxon, 2004; Louis et al., 2006; Axelrad et al., 2008; Handforth, 2012; Buijink et al., 2015; Zhang and Santaniello, 2019; Handforth and Lang, 2021). Two mechanisms have been postulated for the emergence of body tremor in ET: the inferior olive (IO) hypothesis and the cerebellar degeneration hypothesis (see Handforth and Lang, 2021 for a comprehensive review). The IO hypothesis posits that abnormal enhancement of the IO subthreshold oscillations generates suprathreshold synchronized oscillations in Purkinje cells (PCs) and in neurons of the cerebellar nuclei (CN) which subsequently produce body tremor (De Montigny and Lamarre, 1973; Llinas and Volkind, 1973; Elble, 1998; Deuschl and Elble, 2000). The cerebellar degeneration hypothesis posits that partial PC loss together with morphological and connectivity changes in the cerebellar cortex and the CN underlie the emergence of body tremor (Louis et al., 2007; Erickson-Davis et al., 2010; Kuo et al., 2011; Yu et al., 2012; Babij et al., 2013; Louis et al., 2014; Pan et al., 2020).

Pan et al. (2020) generated a mouse model displaying synaptic pruning deficits of climbing fiber (CF) to PC synapses similar to the pathology observed in human ET (Louis et al., 2015). The authors showed that synaptic pruning deficits sufficed to create excessive cerebellar oscillations, which evolved into action tremor that worsened with age. The tremor was eliminated by the silencing of either the IO or the PCs. Similarly, the blockade of PC transmission prevented the induction of body tremor in another animal model of ET; namely, the harmaline model (Brown et al., 2020). Harmaline alters the electrical coupling in the IO and causes synchronized rhythmic firing of its neurons, which generate coherent oscillatory activity in PCs via the CFs that in turn cause rhythmic activity in CN neurons (Lamarre et al., 1971; De Montigny and Lamarre, 1973; Llinas and Volkind, 1973; Batini et al., 1981). This cascade of events causes body tremor (Bernard et al., 1984; Llinas and Muhlethaler, 1988; Jacobson et al., 2009; Handforth, 2012; Brown et al., 2020; Baumel et al., 2021). It has been shown that the onset of tremor is coincident with synchronized rhythmic PC activity, and that mimicking this rhythmicity by optogenetic stimulation sufficed to generate tremor in wild-type animals (Brown et al., 2020; Pan et al., 2020). Thus, the IO and the cerebellar degeneration hypotheses appear to draw on similar electrophysiological characteristics irrespective of whether the tremor evolves with age or is induced by harmaline administration.

In a study on freely moving rats we previously reported that CN neurons display highly coherent oscillations during repetitive and non-repetitive voluntary movements (Baumel and Cohen, 2021; Baumel et al., 2021). We also showed that concomitantly with the emergence of body tremor, harmaline enhanced rhythmic activity in single CN neurons; however, the spectral structure of the oscillations was impaired, causing loss of coherence in the majority of simultaneously recorded neuronal pairs (Baumel et al., 2021). Given that harmaline administration generates highly coherent oscillations in the cerebellar cortex of freely moving rats (Jacobson et al., 2009) it is surprising that this accurate spectral structure was not conserved in downstream neurons (Person and Raman, 2012).

The mibefradil derivative NNC 55-0396 (NNC) is a selective T-type calcium channel antagonist (Huang et al., 2004) which was shown to suppress harmaline-induced body tremor (Handforth et al., 2010; Quesada et al., 2011). To elucidate the role of cerebellar oscillations in health and disease we compared activity in CN neurons during intact motor function, harmaline-induced body tremor and after tremor suppression by NNC. In general terms, all the characteristics that change significantly due to the transition from intact motor function to body tremor may cause tremor, but only those features that recover concomitantly with tremor suppression are likely to be essential for intact motor function and as such become potential candidates for therapeutic approaches.

## 2. Materials and methods

### 2.1. Animals

All procedures were approved by the Bar Ilan University Institutional Animal Care and Use Committee and were performed in accordance with the National Institutes of Health guidelines. Data were collected from 18 Long Evans male rats weighing 350–500 g (Harlan, Indianapolis, IN, USA). All 18 rats were included in the control condition, 15 of the rats were included in the Harm condition and 5 rats were included in the Harm+NNC condition. Data from 15 out of the 18 rats recorded in the control condition and from 12 out of the 15 rats recorded in the Harm condition were included in a previous publication (Baumel et al., 2021). All animals were housed two in a cage, separated by a divider after surgery. Animals were maintained on a 12/12 h light/dark cycle, and had ad-libitum access to food and water. Experiments were performed during the light phase.

### 2.2. Surgery

The surgical procedures have been described elsewhere in Baumel et al. (2009), Jacobson et al. (2009), Baumel and Cohen (2021). In brief, 18 rats were initially sedated by 5% isoflurane and then injected intramuscularly with ketamine HCl and xylazine HCl (100 and 10 mg/kg, respectively). Supplementary injections of ketamine and xylazine were given as required. The skull surface was exposed and 1 or 2 craniotomies, slightly larger than the implanted electrodes, were made above the medial (AP: −11 mm, ML: ±1 mm), interposed (AP: −11 mm, ML: ±1.5 mm), or lateral (AP: −11.5 mm, ML: ±3.5 mm; inserted at 20° angle) nuclei (Paxinos and Watson, 2014). Sixteen microwires (35 μm, isonel coated tungsten; California Fine Wire Company) arranged in 4 × 4 arrays or 16 microwires (25 μm, formvar coated nichrome; A-M Systems, Inc.) inserted into a 29 gauge cannula were lowered 3.4–4.5 mm from the surface of the brain and fixed in position using dental cement. Post-operative care included Carprofen administration (5 mg/Kg; subcutaneous) at 24 and 48 h post-surgery. The animal’s state was evaluated based on predefined parameters and additional injections were given accordingly. Rats were given ≥ 1 week to recover prior to recording. Electrode positioning was verified histologically after performing electrolytic lesions. See Table 1 for information about the neurons’ position across nuclei and their likelihood to oscillate.

**TABLE 1:** Position and percent of oscillatory neurons per cerebellar nuclei.

	IP nuclei	Medial nuclei	Lateral nuclei
Control	66 (osc: 14, 21%)	49 (osc: 17, 35%)	17 (osc: 5, 30%)
Harm	69 (osc: 31, 51%)	22 (osc:13, 60%)	12 (osc: 6, 50%)
Harm + NNC	70 (osc: 29, 41%)	2 (osc: 0, 0%)	6 (osc: 3, 50%)

### 2.3. Data acquisition

Neural activity was amplified, band-pass filtered at 150–8000 Hz and sampled at 40 KHz using a multichannel acquisition processor system (MAP system; Plexon Inc). Activity was recorded for 15 min in freely behaving animals. In sessions where multiple single units were recorded, the animal was lightly sedated (5% isoflurane) and then injected intraperitoneally with harmaline HCl (10–15 mg/kg; Sigma-Aldrich). Neuronal activity was recorded for 15 min after the harmaline-induced body tremor stabilized (∼10–20 min). In five animals, NNC 55-0396 (20 mg/kg) was injected intraperitoneally and after a waiting period of 20 min, neuronal activity was recorded for additional 15 min. Offline sorting (offline sorter, Plexon Inc.) was performed on all recorded channels containing units with a signal to noise ratio exceeding 3:1. A unit was considered a single neuron if its waveform generated a distinct cluster in the principal component analysis performed by the software. Only single neurons were included in the dataset and taken for further analysis in MATLAB (R2013b, MathWorks Inc., Natick, MA, USA).

### 2.4. Data analyses

#### 2.4.1. Firing rate

The firing rate was calculated as the total number of spikes elicited by the neuron divided by the duration of the recording period in seconds.

*Coefficient of variation (CV)*: The standard deviation of the interspike interval (ISI) distribution divided by its mean.

*CV2*. Defined as the difference between two consecutive ISIs divided by their mean.

$$C\,V\,2=\frac{2\,\left|I\,S\,I_{i+1}-I\,S\,I_{i}\right|}{I\,S\,I_{i+1}+I\,S\,I_{i}}$$

#### 2.4.2. Burst detection

The probability of encountering an ISI of length x or shorter, given the preceding interval, was calculated using a measure of the instantaneous discharge probability (Pauluis and Baker, 2000) based on the cumulative gamma distribution.

$$p\,\left(x|A,i\,s\,i_{\left(i\right)}\right)=\frac{1}{A^{i\,s\,i_{\left(i\right)}+1}}\,\int_{0}^{x}t\cdot e^{\frac{-t}{i\,s\,i_{\left(i\right)}}}\,dt$$

Burst initiation was said to occur if two consecutive intervals were shorter than the median ISI and the probability of finding intervals of that duration was < 0.05 based on a gamma distribution whose average ISI was equal to the preceding reference interval. The gamma distribution shape parameter, *A* = 2, was chosen based on its best fit to the dataset. Burst termination was determined according to the Poisson surprise method (Legendy and Salcman, 1985; Benhamou et al., 2012).

*Burst index* was defined as the number of spikes occurring within bursts divided by the total number of spikes.

#### 2.4.3. Power spectrum

For each spike train, an autocorrelation was computed using Matlab xcorr function in 5 s sliding windows with a 50% overlap. In each sliding window, the time resolution was 0.001 s and the autocorrelation was calculated up to 1 s time-lag and transformed into the spectral domain using the Fast Fourier Transform. The power was normalized to dB. A neuron was considered oscillatory if its peak exceeded the 95 % confidence interval of the χ^2^ distribution of the mean power in the 30–50 Hz band and was >3 dB above the power in adjacent frequency bins to prevent the inclusion of noisy bins with large power variation in all frequencies (Percival and Walden, 1993). The same criteria were used for detecting oscillatory bins. The bandwidth of the oscillatory process was defined as the width of the power spectral density peak at half amplitude.

#### 2.4.4. The expected probability of co-occurring oscillatory bins

The probability of each neuron to oscillate was defined as the fraction of oscillatory bins out of the whole recording. The expected probability of two neurons to co-oscillate assuming independent processes was calculated as the product of the probability of each neuron to oscillate.

#### 2.4.5. Coherence

Coherence was calculated by dividing the square of the absolute value of the cross spectrum by the product of the power spectrum of the two neurons. Coherence was computed for time bins in which significant power was obtained in both neurons. The significance of pairwise coherence was assessed independently for each pair of simultaneously recorded neurons by calculating confidence intervals using a bootstrap technique. Specifically, the pairwise coherence was recalculated after randomly assigning a phase difference for each bin in which both neurons oscillated. Confidence intervals were calculated from the distribution of 200 repetitions over the artificially calculated coherence values. If the coherence value exceeded the confidence intervals set at *p* < 0.01, the coherence between the pair of neurons was considered significant.

#### 2.4.6. Statistics

All the data are presented as mean ± SEM unless specified otherwise. The Kruskal–Wallis test for non-parametric distributions was used to statistically compare data between the experimental conditions using Bonferroni correction for multiple comparisons and alpha set to 0.01, unless specified otherwise.

## 3. Results

### 3.1. Suppression of the harmaline-induced body tremor by NNC was not accompanied by restoration of firing pattern properties of single CN neurons

**FIGURE 1:**
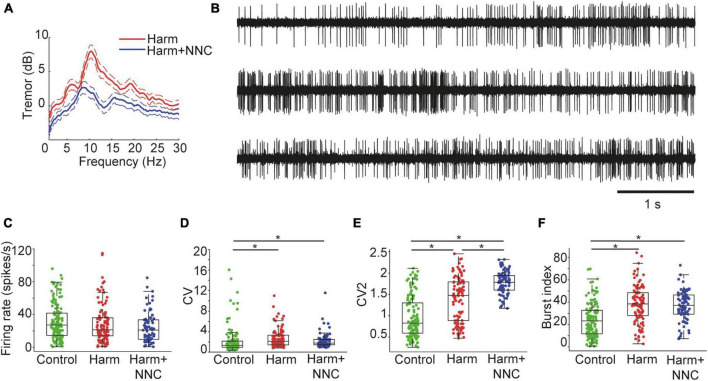
Suppression of the harmaline-induced body tremor by NNC was not accompanied by restoration of firing pattern properties of single CN neurons. **(A)** Power spectral densities of the measured tremor during harmaline application (red trace) and after NNC administration in harmaline treated animals (blue trace). Dashed lines represent the 95th percentile confidence intervals. **(B)** An example of a CN neuron recorded under control (top), Harm (middle), and Harm+NNC (bottom trace) conditions. The neuron displayed diverse firing patterns under all conditions. **(C–F)** Population firing characteristics of CN neurons recorded under control (green), Harm (red), and Harm+NNC (blue) conditions. Shown are firing rates **(C)**, CV **(D)**, CV2 **(E)**, and burst index **(F)**. Boxplots denote the population medians with the edges at the 25th and 75th percentiles. The whiskers extend to the most extreme data point within 1.5 times the interquartile range. Each neuron is represented by a colored dot. Black asterisks denote a significant difference between groups (*p* < 0.01, Bonferroni corrected).

Systemic harmaline administration (intraperitoneal injection, 10–15 mg/Kg) induced severe body tremor that stabilized within 15 min from injection time. The tremor severity was assessed by a wireless accelerometer placed on the animal’s back and by applying power spectrum analysis to the measured signal. The harmaline-induced tremor was characterized by a substantial peak at 10 Hz in the accelerometer power spectrum (Figure 1A, red curve, *n* = 5). Injecting harmaline treated animals with the highly selective T-type calcium channel blocker NNC 55-0396 (NNC, 20 mg/Kg) attenuated the tremor, as reflected in the reduced peak amplitude in the accelerometer power spectrum (Figure 1A, blue curve; mean peak reduction of 38.51 ± 12.17 % in the tremor frequency). This outcome allowed us to characterize neuronal activity recorded during the three conditions: (1) intact motor function in freely moving animals (control), (2) severe body tremor induced by harmaline injection (Harm), and (3) partial recovery of motor function after NNC administration in harmaline treated animals (Harm+NNC). By comparing the activity of cerebellar nuclei (CN) neurons across these conditions, we were able to capture the electrophysiological properties that may account for harmaline-induced body tremor.

We recorded a total of 172 well-isolated single units from the CN of Long-Evans male rats. Of these neurons, 132 were recorded from control animals (*n* = 18), 103 were recorded during the Harm condition (*n* = 15 animals), and 78 of the neurons were recorded during the Harm+NNC condition (*n* = 5 animals). Note that 43 of the neurons were recorded under all three conditions (control, Harm and Harm+NNC). Observation of activity traces of CN neurons revealed diverse firing patterns under each of these conditions (see Figure 1B for representative short activity segments of a neuron recorded during all conditions). We previously reported that harmaline application was accompanied by alterations in several characteristics of single unit activity. Specifically, the global and local coefficients of variation of the interspike interval distributions (CV and CV2, respectively) and the neurons’ tendency to release bursts of activity (burst index, see Methods) significantly increased under harmaline as compared to the controls, whereas the average firing rate did not change across conditions (Baumel et al., 2021). Repetition of this analysis on data from all conditions and inclusion of more neurons in each condition yielded a similar outcome to that reported in Baumel et al. (2021) for the Harm and control conditions, and revealed that the Harm+NNC condition was more similar to the Harm condition than to the control condition in terms of the firing characteristics of the CN neurons. Specifically, the average firing rate of the neurons did not change significantly across conditions [Figure 1C, Kruskal–Wallis (KW) test, χ^2^ = 6.34, *df* = 2, *p* = 0.04], whereas the CV, the CV2 and the burst index were significantly altered (Figure 1D, CV: KW test, χ^2^ = 29.73, *df* = 2, *p* = 3.5e^–7^; Figure 1E, CV2: KW test, χ^2^ = 102.73, *df* = 2, *p* = 4.9e^–23^; Figure 1F, burst index: KW test, χ^2^ = 52.46, *df* = 2, *p* = 4.1e^–12^). Interestingly, while *post-hoc* testing showed that the CV and burst index were similar for the Harm and Harm+NNC conditions (*p* = 0.70 and *p* = 1 for CV and burst index, respectively), the CV2 values measured during Harm+NNC were significantly higher not only relative to the controls but also relative to the Harm condition (control to Harm: *p* = 1.6e^–8^, control to Harm+NNC: *p* = 8.5e^–23^, and Harm to Harm+NNC: *p* = 4.1e^–5^).

Thus, treating the harmaline-induced body tremor with NNC suppresses the tremor without restoring firing properties in single CN neurons such as the CV, CV2 and burst index to their values obtained in the control condition. The fact that the improvement in motor function was not accompanied by a convergence of firing properties of CN neurons to their control condition values suggests that these features may have contributed to the emergence of body tremor but they are less likely to account for its suppression.

### 3.2. Suppression of harmaline-induced body tremor by NNC was accompanied by partial recovery of CN neurons’ oscillation characteristics

**FIGURE 2:**
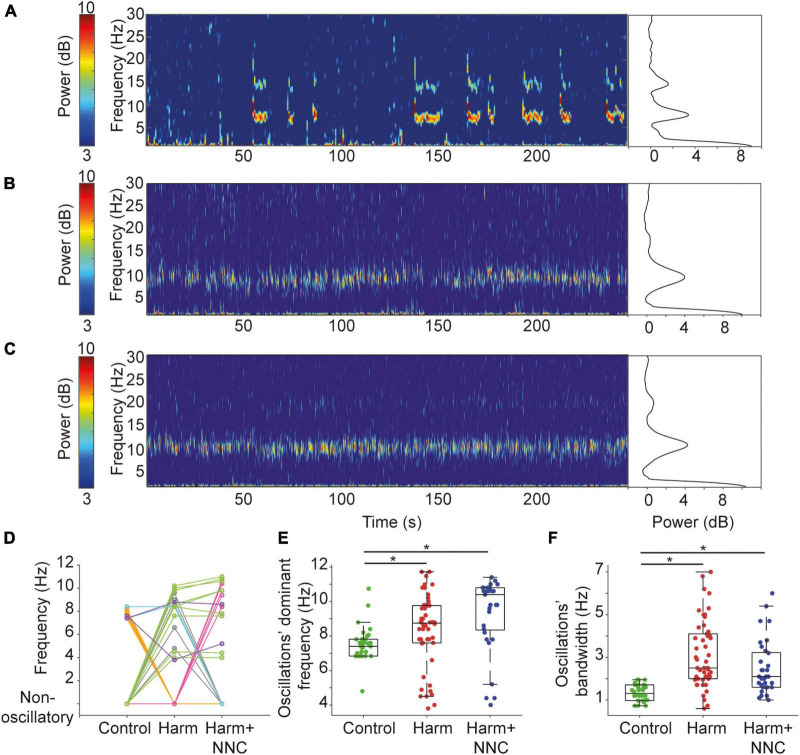
Suppression of harmaline-induced body tremor by NNC was accompanied by partial recovery of CN neurons’ oscillation characteristics. **(A)** A spectrogram of a CN neuron recorded from a freely behaving rat (control condition) showing intermittent oscillatory epochs in the theta range (left). Color bar denotes the spectrogram power in dB. The corresponding average power spectral density (PSD) is shown on the right. **(B)** Same as panel **(A)** for a neuron recorded under the Harm condition. The corresponding PSD includes data from the whole recording of 500 s. **(C)** Same as panel **(A)** for a neuron recorded under the Harm+NNC condition. The corresponding PSD includes data from the whole recording of 560 s. **(D)** Transition between conditions map showing the dominant oscillation frequencies of neurons recorded under all conditions that oscillated during at least one of the conditions (*n* = 32). Different colors identify groups of neurons with a similar transition pattern (see text for color map). **(E)** The dominant oscillation frequency distributions of oscillatory CN neurons under control, Harm and Harm+NNC conditions. Boxplots denote the population medians with the edges at the 25th and 75th percentiles. The whiskers extend to the most extreme data point within 1.5 times the interquartile range. Each neuron is represented by a colored dot. Black asterisks denote a significant difference between groups (*p* < 0.01, Bonferroni corrected). **(F)** The oscillations’ bandwidth distributions of oscillatory CN neurons under control, Harm and Harm+NNC conditions. Boxplot conventions as in panel **(E)**.

We showed elsewhere that during voluntary movement, CN neurons tend to oscillate intermittently in the theta band frequency (Baumel and Cohen, 2021; Baumel et al., 2021) and that the oscillation characteristics are significantly altered after the induction of body tremor by harmaline (Baumel et al., 2021). Figures 2A–C shows example spectrograms and the corresponding power spectra of CN neurons that oscillated in the theta band frequency in the control (Figure 2A), Harm (Figure 2B), and Harm+NNC (Figure 2C) conditions. To determine if the tendency of a neuron to oscillate depended on whether or not it oscillated during the other conditions, we analyzed the 43 neurons that were recorded in all three conditions (Figure 2D). Eleven (26%) of the neurons did not oscillate during any of the conditions. Out of the remaining neurons, the majority (56%, 18/32) oscillated during one condition only: 7 during control (Figure 2D, orange), 5 during Harm (Figure 2D, gray) and 6 during Harm+NNC (Figure 2D, pink), 31% (10/32) oscillated during both Harm and Harm+NNC (Figure 2D, green), 6% (2/32) oscillated during both control and Harm (Figure 2D, light blue), 6% (2/32) oscillated during all conditions (Figure 2D, purple) and none oscillated during control and Harm+NNC. The fact that different neurons, recorded primarily from the IP nucleus, oscillated during the different conditions and that there was no overlap between cells that oscillated in only the control and the Harm+NNC conditions suggests that the identity of the oscillatory neurons within the recorded population was likely not a major factor in the emergence of body tremor in this study.

We then tested whether the oscillation characteristics during the Harm+NNC condition were more similar to those measured during the Harm condition or to those measured during the control condition by repeating the previously reported analysis on the extended data from all conditions. First, we compared the fraction of CN neurons that oscillated in the theta band frequency and the time spent in oscillations relative to the total recording time across conditions. The fraction of oscillatory neurons was significantly different across conditions (36/132 (27.3%), 50/103 (48.5%) and 33/78 (42.3%) in control, Harm and Harm+NNC conditions, respectively; χ^2^ test, χ^2^ = 11.91, *df* = 2, *p* = 0.003). *Post-hoc* comparisons showed that more neurons oscillated after the harmaline injection relative to the control (χ^2^ = 11.28, *df* = 1, *p* = 0.0008); however, the fraction of oscillatory neurons in the Harm+NNC condition did not differ significantly from either the Harm or the control conditions (control vs. Harm+NNC, *χ*^2^ = 5.02, *df* = 1, *p* = 0.03; Harm vs. Harm+NNC, χ^2^ = 0.70, *df* = 1, *p* = 0.40). The time CN neurons spent oscillating relative to the total recording time was significantly different across conditions (38.7, 54.3, and 44.2% for control, Harm and Harm+NNC, respectively; KW test, χ^2^ = 10.45, *df* = 2, *p* = 0.005). *Post-hoc* comparisons showed that as reported previously, under harmaline, CN neurons tended to oscillate during larger portions of the recording time (*p* = 0.004); however, after NNC administration, the time the neurons spent oscillating did not differ significantly from either the Harm or the control conditions (control vs. Harm+NNC, *p* = 0.61; Harm vs. Harm+NNC, *p* = 0.24).

Next, we measured the dominant frequency and bandwidth of the oscillations and compared them across conditions. Both parameters significantly differed across conditions [dominant frequency: KW test, χ^2^ = 25.72, *df* = 2, *p* = 2.6e^–6^ (Figure 2E); oscillation bandwidth: KW test, χ^2^ = 47.12, *df* = 2, *p* = 5.8e^–11^ (Figure 2F)]. However, unlike the parameters analyzed above, *post-hoc* comparisons showed that the dominant frequencies during the Harm and the Harm+NNC conditions (8.35 ± 0.30 and 9.33 ± 0.37 Hz, respectively) were significantly higher than those measured during control condition (control: 7.50 ± 0.16; control vs. Harm: *p* = 0.0032, control vs. Harm+NNC: *p* = 1.6e^–6^ and Harm vs. Harm+NNC: *p* = 0.08). Similarly, the oscillation bandwidths during the Harm and the Harm+NNC conditions (3.13 ± 0.22 and 2.50 ± 0.22 Hz, respectively) were significantly broader than those measured during control condition (control: 1.34 ± 0.06; control vs. Harm: *p* = 3.3e^–11^, control vs. Harm+NNC: *p* = 2.9e^–5^, and Harm vs. Harm+NNC: *p* = 0.19).

Thus, concomitantly with tremor suppression by NNC application, the prevalence of oscillatory neurons and the time spent oscillating were partially restored, thus suggesting that these features are indicative of the current state of the tremor, or that they actively contribute to tremor emergence and its suppression. By contrast, the dominant frequency and bandwidth were not restored thus demonstrating that these characteristics of single CN neurons are not likely to account for the emergence of body tremor or its suppression.

### 3.3. Suppression of the harmaline-induced tremor by NNC significantly improved pairwise coherence in CN neurons

In a previous study on freely moving rats, we reported that pairs of simultaneously recorded CN neurons tended to oscillate at the same time and frequency while maintaining a constant phase lag, thus resulting in high coherence despite recurring intermissions in the oscillations (Baumel et al., 2021). Harmaline application disrupted the precision of the oscillations: not only did the likelihood of simultaneously recorded neurons to oscillate at the same time decrease and neurons could oscillate at different dominant frequencies, but the coherence of pairs that oscillated at the same time and frequency diminished (Baumel et al., 2021). In the current study we aimed to characterize the influence of NNC application on (1) the tendency of CN neurons to coordinate oscillation epochs, (2) the deviation in the dominant frequency of the oscillations within pairs, and (3) the neurons’ ability to maintain a constant phase lag when oscillating at the same time and frequency.

**FIGURE 3:**
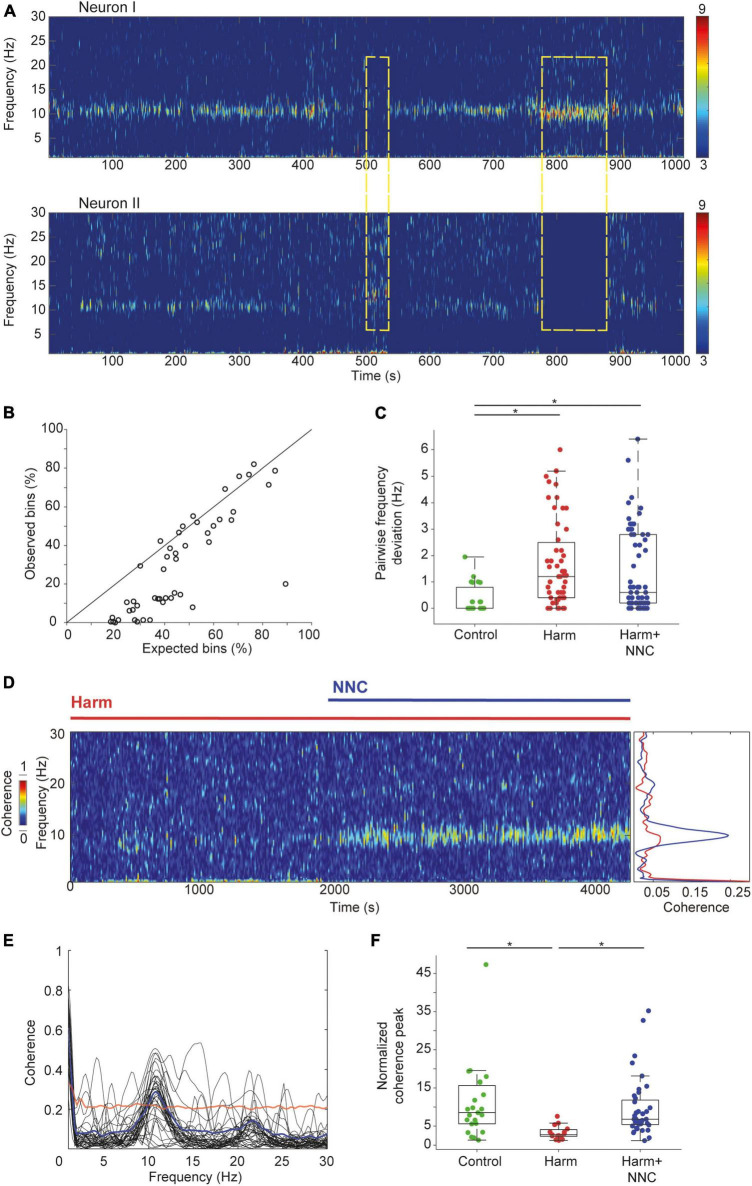
Suppression of the harmaline-induced tremor by NNC significantly improved pairwise coherence in CN neurons. **(A)** An example of two oscillatory CN neurons recorded simultaneously under Harm+NNC condition. Dashed yellow rectangles depict epochs of oscillatory activity in one of the cells but not in the other. **(B)** The expected (abscissa) vs. the observed (ordinate) co-oscillating bins under Harm+NNC condition (*n* = 66 pairs). The diagonal line indicates equal values. **(C)** Distributions of the absolute difference between the dominant oscillation frequencies for all pairs of simultaneously recorded CN neurons under control (*n* = 23), Harm (*n* = 51), and Harm+NNC (*n* = 66) conditions. Boxplots denote the population medians with the edges at the 25th and 75th percentiles. The whiskers extend to the most extreme data point within 1.5 times the interquartile range. Each neuron is represented by a colored dot. Black asterisks denote a significant difference between groups (*p* < 0.01, Bonferroni corrected). **(D)** Time resolved (left) and mean (right) coherence values calculated between two oscillatory CN neurons under Harm (red) and Harm+NNC conditions (blue). **(E)** Oscillation coherence calculated for all neuronal pairs displaying co-oscillating bins with frequency deviation < 1 Hz (*n* = 40 pairs). Thick blue line is the coherence averaged across all pairs. The red line marks the average 99th percentile confidence interval. **(F)** The distribution of the ratio between the peak coherence in the theta range and the mean coherence in the 30–50 Hz range calculated separately for the control (green), the Harm (red), and the Harm+NNC (blue) conditions. Boxplot conventions as in panel **(C)**.

Overall, we recorded 51 and 66 neuronal pairs in the Harm and the Harm+NNC conditions, respectively. The number of neuronal pairs recorded in the control condition (*n* = 23) did not change from that reported in our earlier study despite the increase in the total number of recorded neurons from 115 to 139 (Baumel et al., 2021). First, we calculated the likelihood of neuronal pairs to co-oscillate and compared the outcome to that calculated assuming that these oscillations originated from independent processes (see section “2. Materials and methods”). During control condition, the tendency of CN neurons to co-oscillate was significantly better than chance (as reported in Baumel et al., 2021) with an Observed probability: 36.27 ± 2.48% and an Expected probability: 22.50 ± 2.17%; paired *t*-test, *df* = 22, *t* = 10.95, *p* = 2.2e^–9^). Repetition of this analysis on the extended data during the Harm condition showed that the probability of two neurons to oscillate at the same time did not differ significantly from that calculated assuming independent processes (Observed probability: 43.23 ± 2.74%; Expected probability: 46.97 ± 2.41%; paired *t*-test, *df* = 50, *t* = −2.09, *p* = 0.04). Figure 3A depicts an example of two simultaneously recorded neurons that oscillated in the theta band frequency during the Harm+NNC condition. Throughout the session, the neurons displayed epochs of asynchronous oscillations (Figure 3A, yellow rectangles). Surprisingly, in the Harm+NNC condition, this occurred often, yielding a significantly worse probability than chance that two simultaneously recorded neurons would oscillate at the same time (Figure 3B; Observed probability: 25.86 ± 3.05%; Expected probability: 42.65 ± 2.21%; paired *t*-test; *t* = 10.13, *df* = 65, *p* = 5.2e^–15^). The absence of neuronal co-occurring oscillations after tremor suppression by NNC suggests that this feature does not underlie the emergence of body tremor, however, it may contribute to its suppression.

Next, we compared the fraction of neuronal pairs that oscillated at similar dominant frequencies (<1 Hz deviation) and the average difference in their dominant frequencies across conditions. The fraction of neuronal pairs that oscillated at a similar dominant frequency differed significantly across conditions [23/23 (100%), 26/51 (51%) and 40/66 (61%) for control, Harm and Harm+NNC conditions, respectively; χ^2^ test, χ^2^ = 16.9, *df* = 2, *p* = 0.0002]. *Post-hoc* comparisons showed that the control condition was significantly higher than both the Harm and Harm+NNC conditions whereas the latter two conditions were similar (χ^2^ test, χ^2^ = 1.08, *df* = 1, *p* = 0.29). The average deviation in the dominant frequencies of pairs of oscillating neurons significantly differed across conditions (Figure 3C; mean absolute frequency deviation: 0.35 ± 0.11 Hz, 1.70 ± 0.23 Hz and 1.38 ± 0.19 Hz for control, Harm and Harm+NNC, respectively; KW test, χ^2^ = 18.23, *df* = 2, *p* = 0.0001). *Post-hoc* comparisons showed that the average frequency deviation within pairs was significantly higher in both the Harm and Harm+NNC conditions relative to controls (control vs. Harm: *p* = 0.0001; control vs. Harm+NNC: *p* = 0.003), whereas the pairwise frequency deviation in the Harm+NNC condition did not differ significantly from the Harm condition (Harm vs. Harm+NNC, *p* = 0.41). These results replicate those reported in Baumel et al. (2021) for the control and Harm conditions, and further demonstrate that the fraction of neuronal pairs oscillating at a similar dominant frequency and the frequency deviation between pairs remained similar to the Harm condition and did not improve with tremor suppression.

Finally, we compared how well simultaneously recorded neurons that oscillated at the same time and frequency maintained a constant phase lag by calculating their coherence in the theta band frequency across conditions (see section “2. Materials and methods”). Figure 3D shows an example of the coherence calculated for two simultaneously recorded neurons under harmaline and after NNC administration. This example depicts the absence of coherent activity in the Harm condition and its emergence in the Harm+NNC condition. Overall, NNC administration increased the pairwise coherence and reached significance in 32/40 (80%) of the oscillatory neuronal pairs (Figure 3E, bootstrap, *p* < 0.01, see section “2. Materials and methods”). Repetition of the pairwise coherence analysis on the extended data recorded during the Harm condition only showed a significant coherence in a few pairs [4/26 (15%), bootstrap, *p* < 0.01]. The prevalence of pairs displaying coherent activity across conditions was significantly different [control: 20/23 (87%) reported in Baumel et al. (2021); χ^2^ test, χ^2^ = 35.88, *df* = 2, *p* = 2e^–8^]. *Post-hoc* comparisons showed that the loss of coherence in the Harm condition (control vs. Harm: χ^2^ test, χ^2^ = 25.02, *df* = 1, *p* = 5.7e^–7^), was restored by NNC administration (control vs. Harm+NNC: χ^2^ test, χ^2^ = 0.49, *df* = 1, *p* = 0.48; Harm vs. Harm+NNC: χ^2^ test, χ^2^ = 26.54, *df* = 1, *p* = 2.6e^–7^). The strength of the coherence was assessed by calculating the ratio between the coherence peak and the coherence value at a frequency band of 30–50 Hz (Figure 3F) and comparing the outcomes across conditions. This ratio significantly differed across conditions (KW test, χ^2^ = 18.97, *df* = 2, *p* = 7.6e^–5^). *Post-hoc* comparisons showed that the Harm condition differed from both the control and the Harm+NNC conditions (control vs. Harm: *p* = 0.0002, control vs. Harm+NNC: *p* = 0.99 and Harm vs. Harm+NNC: *p* = 0.0002). The fact that pairwise coherence in CN neurons was the only feature that was completely restored to its control condition values after NNC administration strongly suggests that this feature is essential for intact motor function.

## 4. Discussion

We previously showed that during voluntary movement, CN neurons display intermittent oscillations with a precise spectral structure (Baumel and Cohen, 2021). This accurate spectral structure breaks down during the emergence of body tremor after harmaline administration (Baumel et al., 2021). The findings here extend these results by showing how the spectral structure of the oscillations changes with improvement in motor function by the administration of a specific calcium channel blocker NNC (Handforth et al., 2010; Quesada et al., 2011). NNC administration partially suppressed the harmaline-induced body tremor in freely moving rats, thus allowing us to record single CN neurons during intact motor function, during ET-like body tremor, and during partial recovery of motor function, and to compare electrophysiological characteristics across conditions. Tremor suppression did not affect the firing properties of single CN neurons which remained similar to those measured during full-blown tremor. In addition, although tremor suppression did not affect the dominant frequency and bandwidth of the oscillations relative to harmaline, it partially restored the fraction of CN neurons that oscillated in the theta band frequency and the time spent in oscillations relative to the total recording time. Importantly, the identity of the oscillatory neurons did not correspond to what was detected during intact motor function or during harmaline, thus suggesting that the occurrence of body tremor is not the outcome of a specific population of oscillatory neurons. Analysis of the interactions between pairs of CN neurons showed that the tendency of neuronal pairs to co-oscillate worsened with tremor suppression even relative to the harmaline condition. Moreover, compared to harmaline, tremor suppression did not increase the fraction of neurons oscillating at the same frequency or the frequency deviation between simultaneously recorded neuronal pairs. Finally, the most striking impact of tremor suppression was complete restoration of the ability of neuronal pairs to maintain a constant phase lag during co-occurring oscillations in terms of the fraction of pairs with coherent oscillations and the coherence power. This may imply that for intact motor function, co-oscillating neurons in the CN must maintain an accurate spectral structure.

It has been claimed that the convergence of antiphase oscillations from a range of structures along the movement production network, including the cerebellum, underlie the cancellation of natural oscillations at the motoneuron level, thereby reducing physiological tremor and improving movement precision (Williams et al., 2010). The fact that concomitantly with tremor suppression CN neurons restored oscillation coherence is consistent with this hypothesis and further emphasizes the need to have an accurate spectral structure for intact motor function.

Many studies have reported the occurrence of highly synchronized, coherent oscillations in the theta band frequency in the cerebellar cortex during harmaline-induced body tremor (Llinas and Volkind, 1973; Milner et al., 1995; Jacobson et al., 2009; Park et al., 2010). Reports have indicated that opto-stimulation of the PC—CN synapse in a manner similar to that expected from excessive oscillations in large populations of PCs sufficed to cause body tremor in wild type mice (Brown et al., 2020; Pan et al., 2020). This outcome, together with the observation that CN neurons display oscillations during body tremor led to the speculation that, like in PCs, CN neurons should display coherent oscillations (Zhang and Santaniello, 2019; Brown et al., 2020; Handforth and Lang, 2021). Nevertheless, despite the fact that PCs provide the largest input to the CN (Chan-Palay, 1973; Mezey et al., 1977; Palkovits et al., 1977; de Zeeuw and Berrebi, 1996), it remains unclear why the precise spectral structure becomes aberrant when conveyed to the CN. While other factors may have contributed to this faulty integration of information by CN neurons, the fact that opto-stimulation of CN afferents originating solely from PCs sufficed to generate body tremor suggests that the source is internal to CN neurons, and may possibly involve a shift from their normal dynamic range. Moreover, harmaline administration in animals lacking PC GABA neurotransmission onto CN neurons resulted in little to no tremor (Brown et al., 2020), suggesting that limiting the PCs’ synaptic release enabled CN neurons to respond within their normal dynamic range. It remains to be elucidated whether tremor suppression by NNC directly alters the spectral structure in CN neurons, or indirectly via the IO and/or the cerebellar cortex.

Oscillation characteristics such as the prevalence of oscillatory neurons and the time spent in oscillations were partially restored by NNC administration. We can only speculate that these features may be correlated with the amplitude of tremor suppression and that usage of higher dosages of NNC would have yielded more pronounced tremor suppression concomitantly with more pronounced recovery of these oscillation characteristics. Additional methods are required for determining whether these features are indicative of tremor severity or whether they actively contribute to tremor emergence and its suppression.

The neural mechanism leading to tremor suppression by NNC is unknown. One possibility would be that restoration of the spectral structure in CN neurons enabled these neurons to regain their dynamic range, and rather than exacerbating erratic oscillations and producing body tremor would now be able to generate a rhythmic pattern at the correct phase and amplitude required for canceling out the physiological tremor as is the case in healthy individuals. Another possibility would involve minimizing the time during which two neurons co-oscillate. If neurons oscillate at different dominant frequencies and/or at the wrong phase lags, their interference may enhance the oscillatory output rather than canceling it out, thus enhancing tremor. Thus, by avoiding conjoint oscillations in CN neurons, the interference in their output is reduced. Our data provide supporting evidence for the existence of both possibilities since the coherence between CN neurons was fully restored, and the tendency of simultaneously recorded neurons to co-oscillate was significantly worse than expected by chance.

Despite the fact that the source of ET remains elusive, understanding its electrophysiological manifestations can facilitate selection of effective treatment. For example, it remains unclear whether the breakdown of the spectral structure in CN neurons described here occurs in other animal models of ET such as hotfoot17 and/or in the human ET. If this is indeed the case, then concentrating efforts on developing methods for retaining the normal dynamic range in CN neurons would be highly beneficial in treating ET.

## Data availability statement

The raw data supporting the conclusions of this article will be made available by the authors, without undue reservation.

## Ethics statement

The animal study was reviewed and approved by the Bar Ilan University Institutional Animal Care and Use Committee.

## Author contributions

YB and DC conceived and designed the experiments. YB performed experiments. YB and HY analyzed the data. YB, HY, and DC interpreted the results of the experiments, prepared the figures, drafted the manuscript, and approved the final version of the manuscript. DC edited and revised the manuscript. All authors contributed to the article and approved the submitted version.
